# Occupational accidents and the use of PPE: a global meta-analysis

**DOI:** 10.3389/fpubh.2024.1368991

**Published:** 2024-06-21

**Authors:** Ginevra Malta, Serena Matera, Fulvio Plescia, Anna Calascibetta, Antonina Argo, Emanuele Cannizzaro

**Affiliations:** ^1^Department of Health Promotion, Mother and Childcare, Internal Medicine and Medical Specialties (PROMISE), Palermo, Italy; ^2^Department of Clinical and Experimental Medicine, Occupational Medicine – University of Catania, Palermo, Italy

**Keywords:** work injuries, work health, PPE, work accidents, occupational, sustainibility, risk, safety

## Abstract

Occupational accidents, despite continuous safety updates, are still a scourge in the occupational and forensic spheres, constituting, among other things, the subject of a large share of litigation. Demographic data can help to understand the areas where the application of health surveillance is lacking. This meta-analysis sets out to analyse data from studies on accidents at work, focusing on the correlation between the areas in which accidents occur and whether or not personal safety equipment is used, in relation to the different regulations in force. For the selection of the data, a systematic review was carried out according to the PRISMA guidelines, with the primary objective of identifying the trend of occupational accidents in specific geographical areas, which differ in terms of the attention paid to preventive aspects. The data we highlighted showed, regarding the type of accident, substantial differences between low-income countries and industrialised countries (stratified according to the Human Development Index) and, an overall indifference as to whether or not individual safety devices were used, revealing that, despite the continuous normative evolution in the field of safety at work, even today, the investigative data on the actual application of the regulations, during accidents at work, is underestimated and little researched.

## 1 Introduction

Accidents at work account for an important share of the disabling and unfortunate outcomes of the overall accidental caseload. These can result from different factors related both to the work environment, inadequate use of safety equipment, unsafe working conditions, and to the individual, fatigue and stress, inadequate training and education, lack of attention or disobedience to safety rules. Even today, in spite of the present deluge of legislation and implementation, they still represent a major public health problem, being responsible for a considerable number of cases of morbidity and mortality worldwide, especially in low- and middle-developed countries (1, 2).

According to the International Labour Organisation (ILO), every year about 2.3 million women and men worldwide succumb to work-related injuries or illnesses; this corresponds to more than 6,000 deaths per day. Worldwide, there are approximately 340 million work-related injuries and 160 million victims of occupational diseases each year (1, 3).

The Occupational Safety and Health Administration (OSHA) and the National Institute for Occupational Safety and Health (NIOSH) are two US agencies that play crucial roles in the promotion of safety and health in the workplace. Although they have similar objectives, their methods of monitoring and intervening in occupational accidents differ in their functions and approaches. OSHA focuses primarily on the regulation and enforcement of occupational safety and health regulations. It uses various methods to monitor occupational injuries, including inspections (which can be initiated as a result of complaints, injuries, referrals or specific inspection programmes targeting high-risk sectors), injury reports and injury prevention programmes promoted by the agency itself. NIOSH, being part of the Centers for Disease Control and Prevention (CDC), is in charge of research and recommendations for the prevention of occupational injuries and illnesses, surveillance (collecting and analysing data on occupational injuries and illnesses to identify trends and areas of risk, including through the use of national databases and industry-specific surveys), and issuing safety recommendations that may also influence OSHA policies and industry practices.

In summary, while OSHA focuses more on enforcement and compliance with occupational safety regulations, NIOSH plays a research role and provides evidence-based recommendations to prevent occupational injuries and illnesses. Both work closely together to improve the safety and health of American workers (4).

The importance of recording the use of Personal Protective Equipment (PPE) in the workplace is underlined by several international occupational health and safety regulations and guidelines. These regulations aim to ensure that workers are adequately protected from the hazards present in their work environments and that there is traceability and accountability in the use of PPE.

The ILO establishes general guidelines for occupational safety and health, including the importance of PPE. Although specifications may vary depending on local legislation, the ILO emphasises the need for a systematic approach to risk assessment and the use of PPE as a control measure when risks cannot be eliminated or reduced sufficiently through engineering or administrative measures.

Studying when and how PPE is used or ignored can help identify gaps in training, safety culture, or PPE availability. This can lead to the adoption of corrective measures to increase the use of PPE.

In order to shed light on the cause and manner of death, a careful analysis of whether or not personal protective equipment was used is essential, together with the subject’s work history, evidence at the scene, and forensic investigation. The investigation of occupational fatalities requires a standardised methodology, the collaboration of experts in several forensic sciences, cross-examination and dialogue between occupational safety experts and laboratory pathologists. However, forensic trauma diagnosis is extremely diverse, with significant differences in work skills and technical developments in different countries.

The socio-cultural context and legislation inevitably influence the rates and methods of occurrence of occupational accidents. Therefore, significant differences can be observed between countries. In industrialised countries, accidents related to work equipment and falls are the most frequent, while in developing countries pesticide poisoning and electrocutions are more common. A common factor is the existence of large-scale industries and smaller factories; between these two, not only the implementation of health surveillance, but the history collection itself is different (1).

Research on the accident patterns and risk factors involved is still inadequate. Moreover, the studies in the literature are often not homogeneous, which leads to inconsistent and unrepresentative socio-demographic and public health analyses. This consideration often stems from the time lapse between injury and recovery, or injury and death, especially when this does not occur immediately and is determined by the possible contribution of supervening causes attributable to lengthy hospitalisation (5).

Although there is no globally uniform standard, it is self-evident that the use of personal protective equipment, in a controlled work environment, is a determinant in the reduction of adverse events (6).

In order to raise the awareness of the community and future political and health strategies on the importance of occupational injury prevention, the authors analysed the works in the scientific literature to emphasise the importance of the association between ‘safety and protection’ for preventive purposes. In particular, they focused on investigations into the use or non-use of PPE during fatal accidents.

## 2 Materials and methods

### 2.1 Protocol

The authors conducted a systematic search of studies published between 2014 and 2020. The protocol of this study was designed following the Preferred Reporting Items for Systematic Reviews and Meta-Analyses (PRISMA) guidelines (7) and using the methodology described in the Cochrane Collaboration Handbook on Systematic Reviews of Health Promotion and Public Health Program (8).

### 2.2 Data sources and search strategy

Records were identified using the PubMed search engine. For the search, MeSH terms and free text words were combined through Boolean operations as follows: (WORK) AND (DEATH) OR (INJURY/INJURIES) OR (P.P.E.) OR (ILLICIT DRUGS) OR (TOXICOLOGY). The search was completed in September 2023.

### 2.3 Inclusion and exclusion criteria

Studies on series of fatal and non-fatal occupational accidents focused on a specific country with data on occupational, anamnestic and social information were included.

The inclusion criteria for the studies were as follows:

- The article was in English.
- The article was original.
- The study covered both fatal and non-fatal occupational accidents, also in the context of a general case study.
- The study included at least 20 patients;
- The study contained data on fatal and non-fatal work-related injuries;
- The study contained data on P.P.E. use and/or alcohol/drug consumption;
- The studies belonged to different groups according to the HDI.

**Figure 1:**
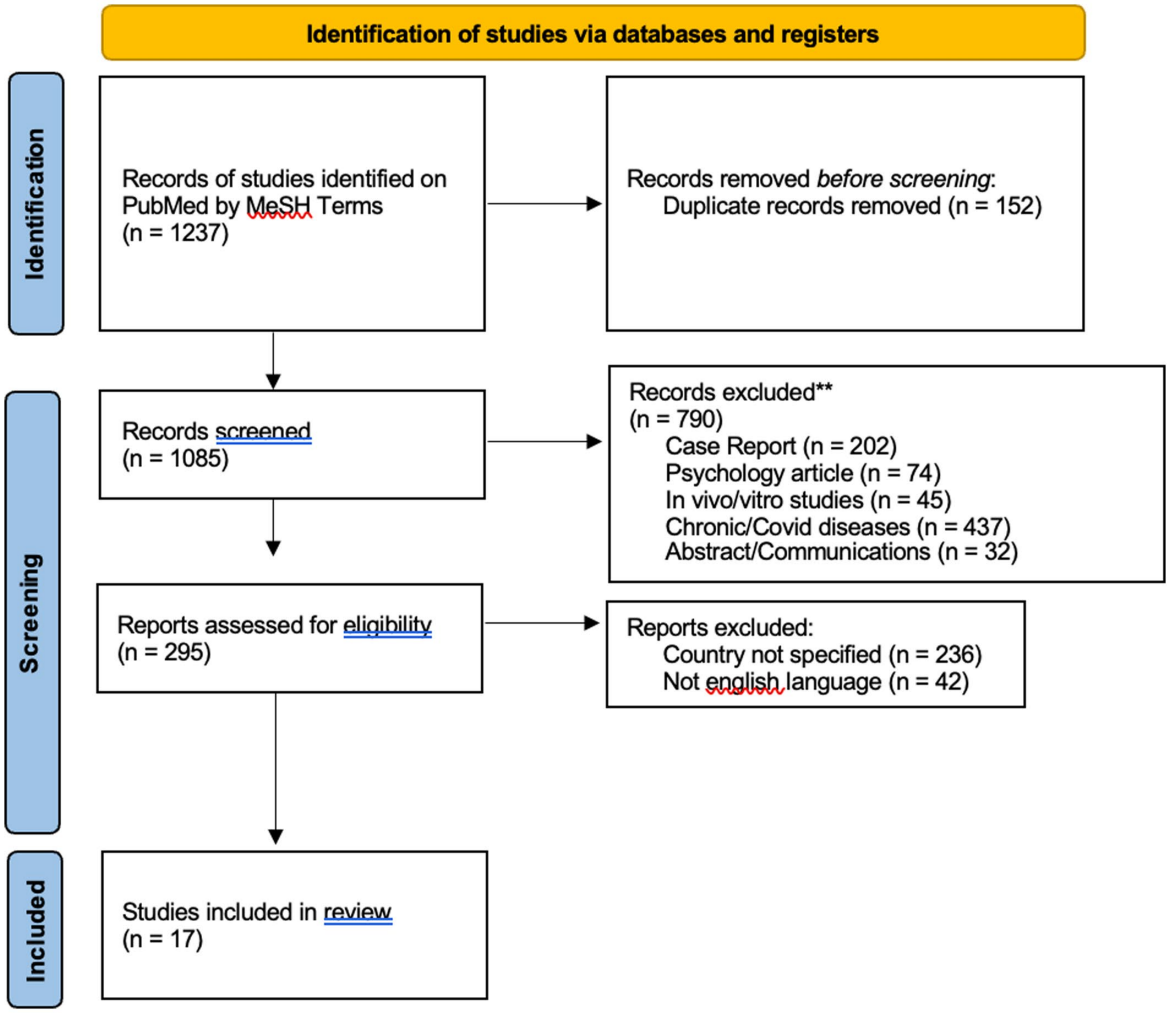
Flow diagram illustrating the search strategy and included and excluded studies in this systematic review.

The following were excluded: psycho-sociological studies, case reports, posters, abstracts and conference communications, non-English articles, *in vivo* and *in vitro* studies, articles that did not relate to a specific country, and articles on occupational diseases and chronic/neoplastic diseases and on COVID 19. A detailed flow chart of the selection process is provided in Figure 1.

### 2.4 Study selection and data collection process

Initially, articles were selected on the basis of title and abstract. Subsequently, a full-text evaluation of the selected studies was performed. Based on the literature search, 18 studies were identified that met three, or more, inclusion criteria.

For each study, two authors (GM, SF) extracted the data using a pre-prepared Excel spreadsheet according to the following variables: year of the study, reference period, country, type of sample (general, workers of specific activity of interest for the selected country), severity of the injury (fatal, non-fatal), no. of work-related injuries, gender (male, female), average age, assessment on the use of personal protective equipment, toxicological investigations.

To begin with, from 1,237 articles, 152 duplicates were removed and 1,085 records were screened. Thus, 967 records were excluded according to the exclusion criteria. After full-text evaluation, a further 110 records were excluded as non-specific. The quality of each study was assessed independently by AC and SF by PICO method. If there was a conflict of opinion on the articles, they were submitted to EC. Finally, 17 articles were included in this review. A detailed flow chart of the selection process is shown in Figure 1.

### 2.5 Study stratification

The countries in the selected studies were stratified according to the Human Development Index (HDI), a macroeconomic development indicator that is a different way of assessing the well-being of a nation, because it takes into account not only the gross domestic product (GDP) *per capita*, but also other societal factors, including life expectancy at birth, the amount of food calories available *per capita*, the availability of drinking water, the literacy rate and schooling rate of the population, access to health services and the degree of political freedom.

HDI is defined as the geometric mean of three basic indices, linked, respectively, to life expectancy, education level and income, which in turn are calculated as follows (9).

Specifically, the three indices reflect:

- the health dimension which is assessed by life expectancy at birth,
- the educational dimension as measured by years of schooling for adults aged 25 and over and expected years of schooling for school-age children,
- the size of the standard of living, which is measured by gross national income *per capita*.

Since the last HDI report, countries are divided into four groups according to the quartile they fall into (9):

- top 25% of countries: very high human development countries
- 25 to 50% of countries: high human development countries
- 50 to 75% of countries: medium human development countries
- last 25% of countries: countries with low human development

## 3 Results

All selected studies are summarised in Table 1.

**Table 1:** Summary of the systematic review.

Group by HDI	Authors/Year	Group of study	Country	Study design	Workers’ Population	Outcome			N. W-RD	% W-RD	Gender		Mean Age	Analysis about use of PPE	Toxicological analyses
						Fatal	Nonfatal	Total			M	F			
1	Errico S. et al. (2022)	General	Italy	Retrospective (2011–2020)	Construction and steel manufacturing industries	47	0	47	47	100	46	1	49,5	Yes	No
1	Perotti S. Russo M.C. (2018)	General	Italy	Retrospective (1982–2015)	Construction, mechanical factory, agriculture, transport, mining, firework manufactories, chemical industry and healthcare workers	426	0	426	426	100	422	4	41	No	Yes
1	Anh Hoang B.S. et al. (2021)	Industry (paint)	USA	Retrospective (1980–2018)	Workers use paint strippers, cleaners, adhesives, and sealants (methylene chloride- containing product)	85	37,201	37,286	74	0,2	67	4	35	Yes	Yes
1	Obeid N.R. et al. (2016)	Falls	USA	Prospective (2000–2010)	Industries and edil workers	423	0	423	54	12,7	324	99	43,5	No	No
1	Ramirez et al. (12)	General	USA (Iowa)	Retrospective (2005–2009)	Agriculture, transport, public safety, industry and mining workers	427	0	427	427	100	384	22	51	No	Yes
1	Antunes et al. (2018)	Tractors drivers	Portugal	Retrospective (2005–2014)	Agriculture workers	3,508	0	3,508	57	1,62	57	0	60	No	Yes
1	Ozer et al. (2014)	Coal mine workers	Turkey	Retrospective (2005–2008)	Coal mine workers	42	0	42	42	100	42	0	32,9	No	Yes
1	Jurek et al. (2017)	Agriculture	Poland	Retrospective (1991–2014)	Agriculture workers	18,935	0	18,935	98	0,52	96	2	47,5	No	Yes
2	Wang L. et al. (2019)	Poisoning	China	Retrospective	Agriculture workers	1968	0	140	56	40	64	29	0	No	Yes
2	Shuiping L. et al. (2014)	Electrocution	China	Retrospective (2008–2017)	Welders, builders, decoration automobile repairmen, machinists, electricians, and polish workers	3,370	0	3,370	30	0,9	30	0	33,08	No	No
2	Cordeiro R. et al. (2017)	General	Brazil	Retrospective (2015)	Workers in the production of industrial goods and services, except machine operators, military. Strict labour accidents. Work-related accidents in traffic. Others	415	0	415	82	19,75	74	8	42,5	No	No
2	AL-Abdallat et al. (2014)	General	Jordan	Review (2008–2012)	Construction workers in addition to governmental, military, security and police force workers	88	0	88	88	100	87	1	32,5	No	Yes
2	Vahabi et al. (2017)	General	Iran	Original Article (2012–2013)	Agriculture, food and tobacco industries, chemical product, electronic and non-electric workers	96	21,388	21,484	96	0,44	2,113	371	33,2	No	Yes
2	Nazanin et al. (2019)	General	Iran	Retrospective (2007–2016)	Agricultural sector (farming, fishing, and forestry), industrial sector (mining, manufacturing, energy production, and construction), and service sector (office workers, public service, transfer, business)	1,079	206,525	207,604	1,079	0,52	203,322	4,281	0	No	No
2	Pouradeli et al. (2022)	General	Iran (Kerman)	Cross-selection study (2012–2016)	Construction simple workers, technical service and office Mining, transportation, agricultural, others	263	1965	2,228	263	11,8	2,181	47	34,5	No	Yes
3	Chittaranjan B. (2019)	Electrocution	India	Retrospective (2002–2017)	Electric and Grinding machine workers	428	0	428	90	21,03	376	52	30,5	No	No
4	Shewijo et al. (2021)	General	Tanzania	Article (2016–2019)	Workers of transportation and storage sector; information and technology; construction and building; and electricity, gas, and steam sectors, teachers, drivers, office workers, and security guards	236	4,342	4,578	236	51,5	217	19	34	No	No

W-RD, work-related death; PPE, personal protective equipment.

### 3.1 Characteristics of articles included in the systematic review

A total of 17 articles that met the selected inclusion criteria were selected.

The countries were then grouped according to their Human Development Index into:

- Group 1: countries with very high HDI: Western Europe, i.e., Italy (no. 2 articles), Portugal (no. 1 article), USA (no. 3 articles), Turkey (no. 1 article), Poland (no. 1 article);
- Group 2: countries with high HDI: China (no. 2 articles), Brazil (no. 1 article), Jordan (no. 1 article), Iran (no. 3 articles);
- Group 3: countries with average HDI: India (no. 1 article);
- Group 4: countries with low HDI: Tanzania (no. 1 article).

**Figure 2:**
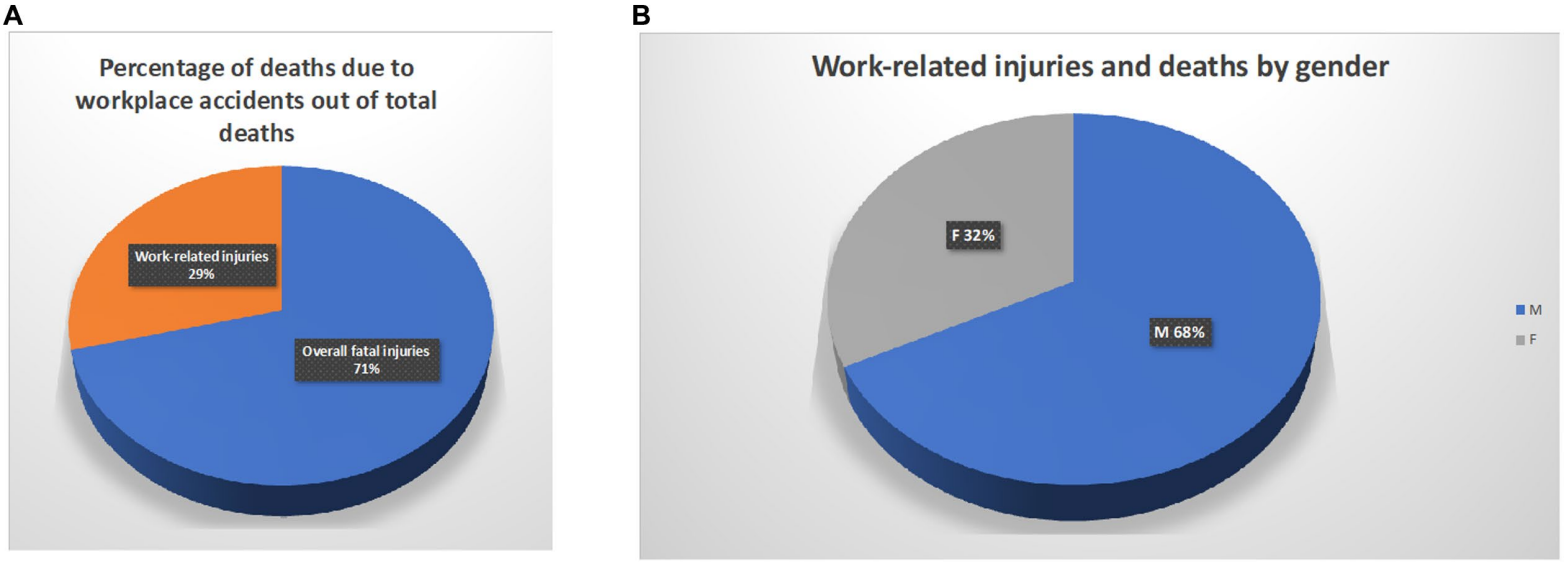
Proportion of work-related deaths in the general population **(A)** and in relation to gender **(B)**. All data relate to the case studies analysed in this study.

Eleven articles were retrospective studies conducted on at least five-year case histories; one study was prospective; one a review; the remainder were classified as original articles. The overall period covered by the studies was 1980-2019.The global case series of the studies included 6,704,622 injuries, of which fatal 6,464,917, non-fatal 241,118, fatal work-related 2,726,397; the estimated global mean age was 34.32 years, with a prevalence of the male gender (1,990,711 men, 951,597 women). Calculating the mean age by gender was not possible as it was not specified in each selected article (Figure 2).

**Figure 3:**
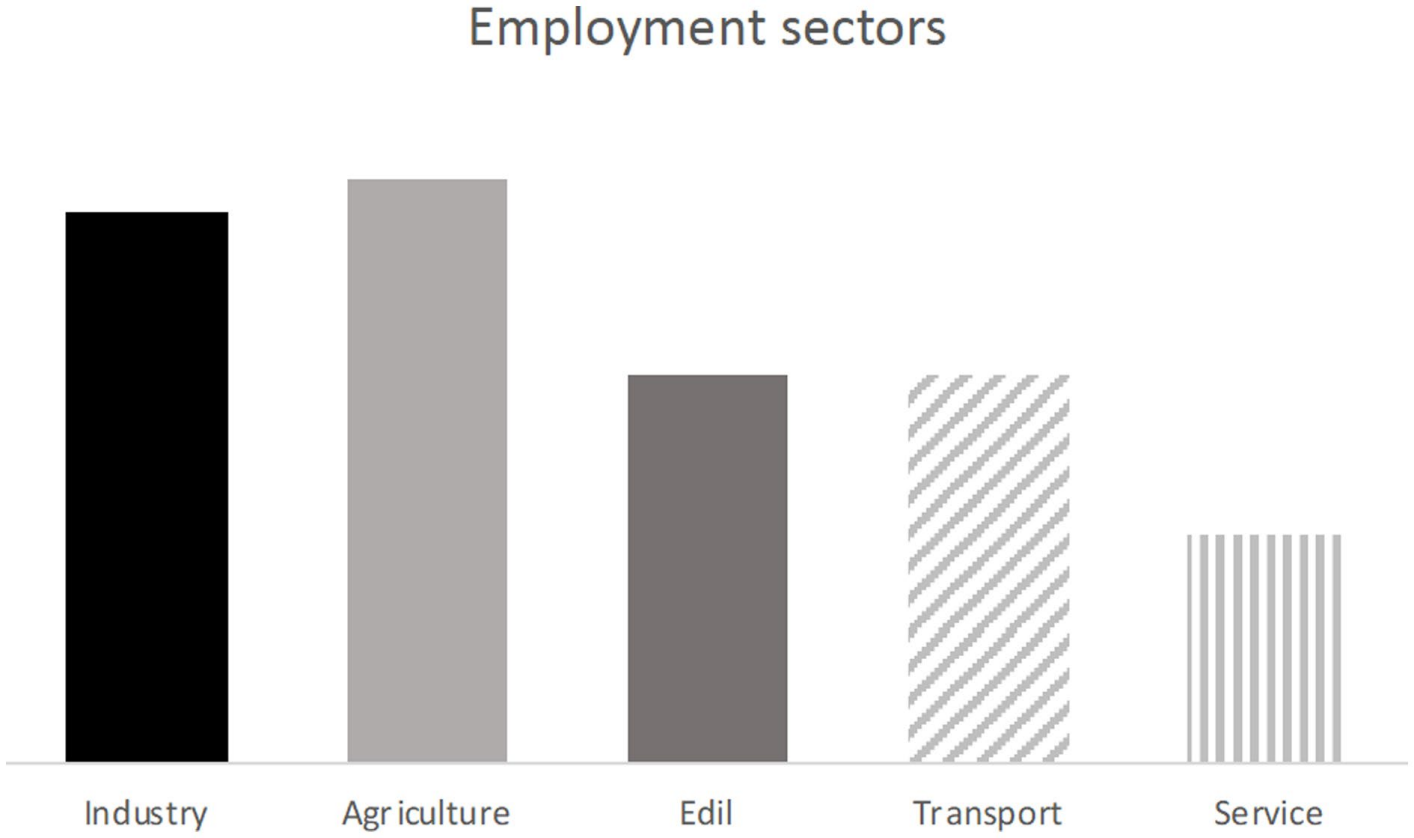
The graph shows the work sectors most affected in work-related fatalities.

From the studies where reported, a binary code database was created to obtain an overall estimate of the occupational sector most involved in accidental events. It was found that the sectors primarily involved are industry and agriculture, followed by construction, transport and finally services (Figure 3).

### 3.2 Statistical analysis

In order to understand the differences that exist in terms of incidence by age, a comparative analysis was conducted for all selected countries using data from studies that sectionalised work-related deaths by age.

In detail, within the sample analysed, a higher number of deaths (84.4%) + in the age group 30–39 (put number not percentage; CI 15.42–125.1) was highlighted. No differences in the number of deaths were highlighted in the 40–49 age group, although a positive trend was found within the 50–59 age group.

**Figure 4:**
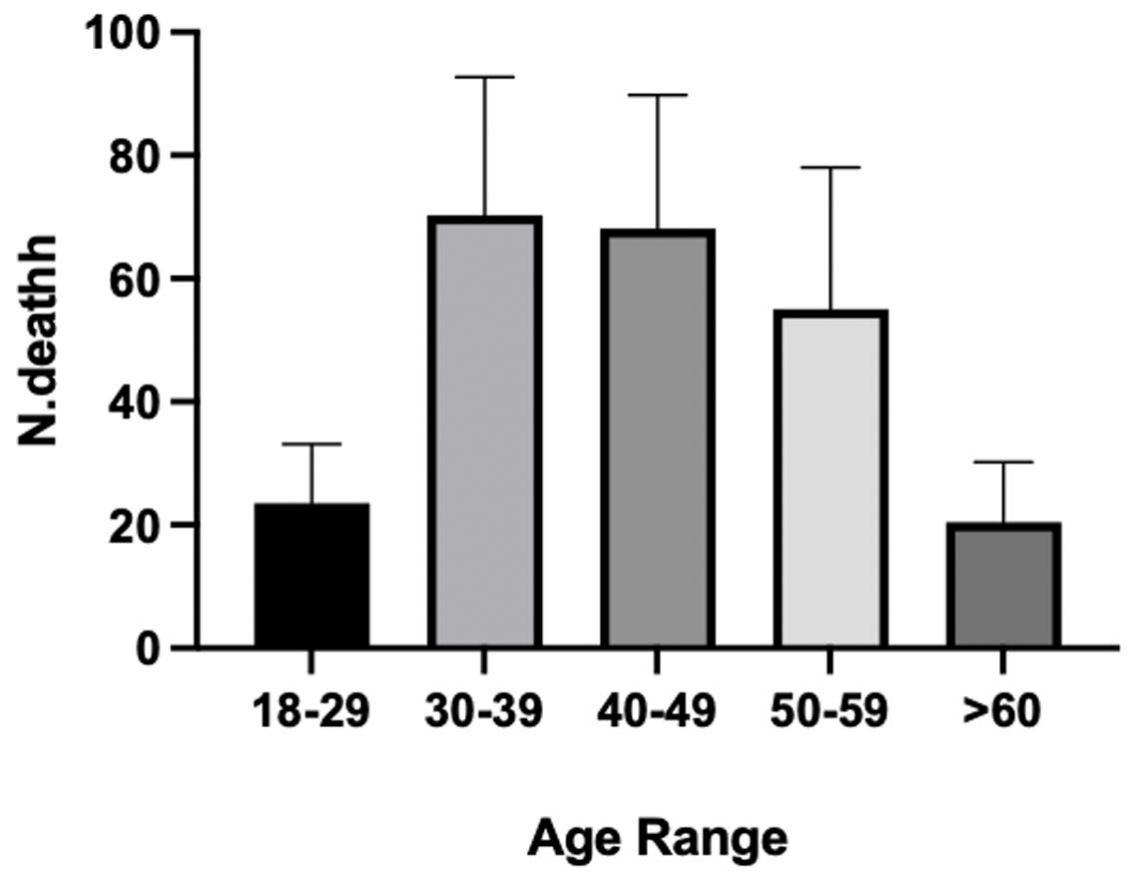
Data on the prevalence of work-related fatalities by age. Age groups are representative of a sample of 1,662 subjects whose gender was numerically specified in the selected studies.

A comparative analysis of the studies containing the age groups by no. of fatalities was carried out, which revealed that the age group most involved in work-related fatal accidents is in the 30–49 age range, compared with the under 30 and over 50 age groups (Figure 4).

In order to understand the incidence of deaths in individual countries by age group (divided into 10 year periods from 18 to 60 years), the chi-square test was performed, which revealed a significant difference within the sample analysed (*p* value <0.05).

**Figure 5:**
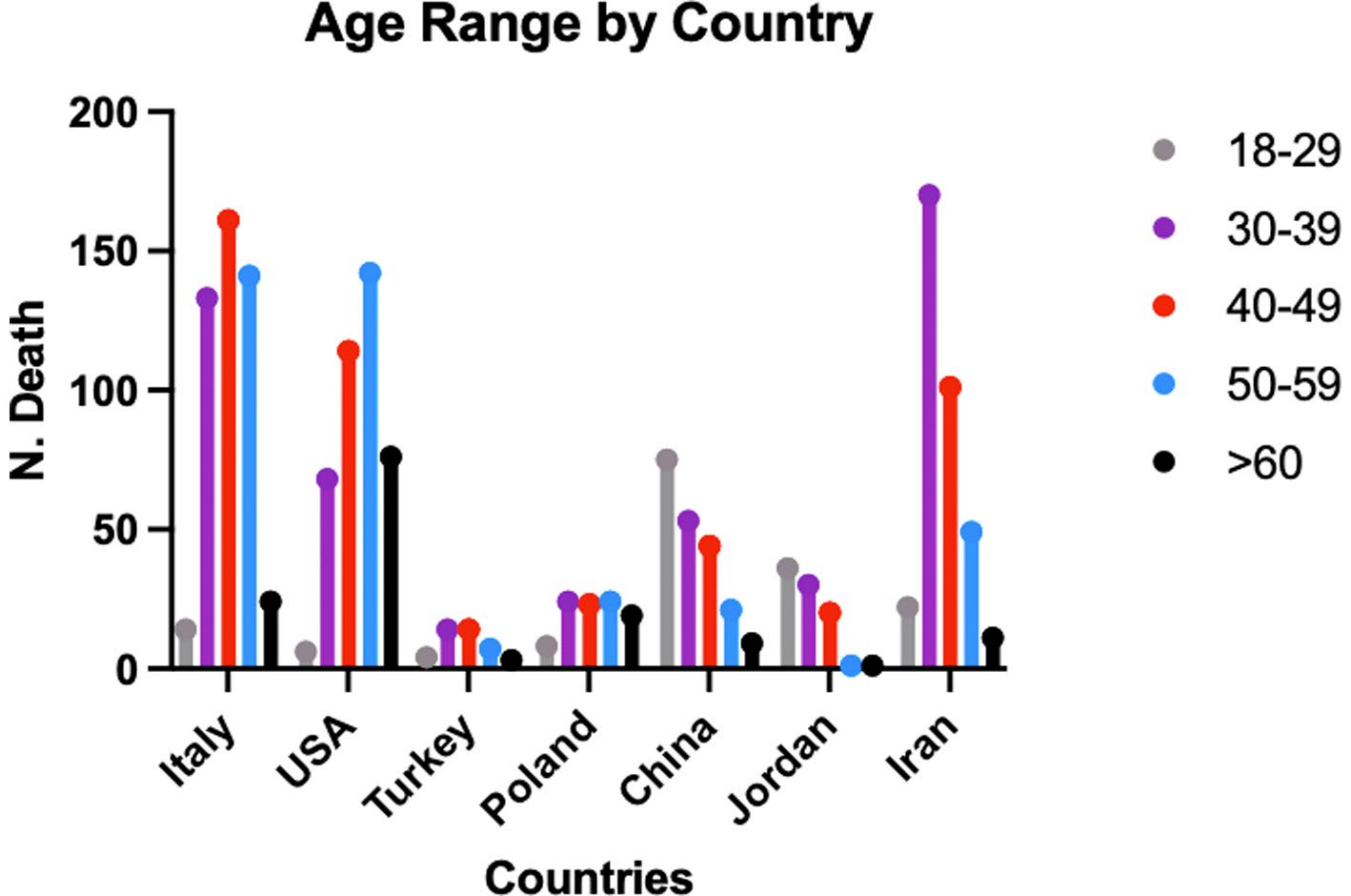
Data on the prevalence of work-related fatalities by age and country from the studies containing the population stratification of the subjects included in the sample.

In demographic terms there was a certain homogeneity in the distribution of fatalities by age group, with a higher prevalence of the involvement of young people in Turkey, Poland (ISU Group 1), China and Jordan (ISU Group 2). In the countries belonging to ISU Group 1, the incidence of fatal accidents in individuals aged between 30 and 49 was statistically significant (Figure 5).

#### 3.2.1 Group 1—very high HDI (Italy, United States, Portugal, Turkey, Poland)

##### 3.2.1.1 Italy

The analysis of the retrospective study conducted by Errico et al., which analysed 47 fatal accidents at work, of which only one case was female, showed that the average age of the deceased was 54.5 years and that in 87.2% of the cases the death was attributable to mechanical trauma related to industrial activities, while in 42.5% of the cases it was precipitation trauma. With regard to the use of PPE, in 97.2% of the cases this was not specified, in 6.4% correct use was reported and in 4.2% incorrect use (10).

In the study conducted by Petrotti et al. (11) out of 426 cases of fatalities ascribed to work-related injuries, 36.62% of the cases occurred within the construction industry, 19.25% due to the use of industrial machinery, 13.5% within agriculture, 5.16% occurred among transporters and, only 0.94% within the chemical industry.

Of the total number of cases, 422 were men and 4 women, with an average age of 27.93% involving subjects under the age of 35. The study, however, did not specify any observations regarding the use of PPE (11).

##### 3.2.1.2 United States

In the United States, Ramirez et al. analysed occupational deaths and selected the cases in which toxicological investigations had been conducted. Of a total of 427 cases, only 280 had been investigated and 61 cases were found to be positive for substances of abuse of recent use. Although the occupational subcategories were analysed, there were no observations in the study regarding the use of PPE (12).

Obeid et al. carried out a prospective study of 423 fall-related deaths, of which 54 were work-related, or 12.76% of the total. The victims consisted of 324 men and 99 women, with an average age of 43.5 years. The 54 work-related cases were all men and the ultimate cause of death was attributed to the polytrauma suffered. With regard to the use of PPE, the authors report, as an indication for preventive purposes, that 4% of work-related deaths were attributable to the improper use of PPE (13).

The retrospective study conducted by Anh Hoang et al. on a total of 6,674 accident cases, of which 85 were work-related (1.28% of the total) and, specifically, 75 were male, 10 female, with an average age of about 35 years. The accidents were related to the use of chemical agents in the context of design. In 31 cases the use of PPE was not specified; in 36 it was reported as correct, in 16 as not being used, and in 2 cases as being used inappropriately (14).

##### 3.2.1.3 Portugal

In the Portuguese territory, the most frequently studied work-related accidents are related to agricultural activities. The study conducted by Antunes et al., examined 3,508 cases of fatalities of which 57 (1.62%) were attributable to the driving of tractors by male workers with an average age of 60 years. This study investigated both the use of personal safety equipment, which was not used in any case, and the toxicological profiles of the victims, which showed that traces of pesticides were present in 1.7 per cent of the subjects. No data on the use of PPE (15).

##### 3.2.1.4 Turkey

In accordance with the typical work activities in the area, Ozer et al. analysed the deaths of mine workers that occurred during the time period 2005–2008 and found 42 fatal cases, in which the average age of the subjects (all male) was 32.9 years. Their study showed the need to implement prevention and safety systems in working environments, especially in private industries, in the absence, however, of precise data on the use of PPE (16).

##### 3.2.1.5 Poland

Jurek et al. conducted a 20-year retrospective study focusing on the sub-population of agricultural workers who suffered fatal accidents and in whom ethyl alcohol intoxication was detected at the same time. The choice was dictated by the assessment of occupational risks. Of a total of 18,935 autopsies, 98 cases of agricultural-related accidents with ethyl alcohol intoxication ranging from 50 mg/dL to >250 mg/dL were selected. However, the use of personal safety equipment was not investigated (17).

#### 3.2.2 Group 2—high ISU (China, Brazil, Iran, Jordan)

##### 3.2.2.1 China

The study conducted by Wang et al., analysed 1968 cases of deaths of which 140 were ascribed to poisoning. Of these, 66.43% (56 cases) were accidental and (40%) work-related, with a male predominance (78 men, 62 women). In 27.1%, the poisoning was related to the use of agrochemicals, in 55.2% pesticides and, in the remaining cases, accidental inhalation of insecticides, herbicides, rodenticides, carbon monoxide and cyanogenic gases and other solvents. The use of appropriate PPE was never investigated (18).

Another type of fatal injury that has been particularly highlighted in China is low voltage electrocution [Shuiping et al., (19)]. Of 3,370 cases that occurred, 0.89% were work-related injuries, suffered by males, with an average age of 33.08 years. The use of appropriate safety equipment to prevent the type of injury was not investigated in detail (19).

##### 3.2.2.2 Brazil

Constituting a current problem in the area of injuries and deaths in the workplace, Cordeiro et al., using special criteria to select the definition of injury/death in the workplace, identified 82 cases, out of 415, of work-related deaths (i.e., 19.76%); 74 subjects were men, 8 women; the average age was 42.5 years in the absence of any specification of the individual age groups involved. Of the 82 cases selected, 62 were regularly employed; 12 were not formally employed; 8 in unknown employment. When assessing the use of licit and illicit drugs among the deceased workers, 50 tested positive for alcohol use in the month prior to death, 28 were smokers, 13 tested positive for drug use and 14 had a known history of problematic alcohol and illicit drug use. Out of the total number of cases, 35 (42.7%) were classified as traffic accidents and were caused by collisions and run-offs on urban roads and motorways. The three (3.7%) work-related injuries classified as other types of work-related injuries were suicides, all committed in the workplace during working hours. In one case, a handyman, chronically exposed to organophosphates, hanged himself while cutting grass in a public garden. In another, an industrial painter, chronically exposed to organophosphate solvents, deliberately threw himself against a bus passing the industry gate as he was leaving work. The third worker was the owner of a small carpenter’s shop who hanged himself in his office during working hours, leaving a letter stating that he could no longer cope with the pressure of his factory’s creditors demanding back payments. Also in this study, the use of PPE was not investigated (20).

##### 3.2.2.3 Jordan

The review conducted by AL-Abdallat et al. examined 88 cases of work-related fatalities in which only one person was female, which is compatible with the culture of the area. The average age was 32.5 years, in the absence of details on the individual age groups involved. Most events were related to falls from height (44.3%), electrocution (17%) and minor events. Toxicological investigations revealed that only one of the cases investigated was positive for alcohol consumption. The possible use of preventive measures was not investigated (21).

##### 3.2.2.4 Iran

A macro-case history of work-related injuries in Iran was analysed by Vahabi et al. in which, of 21,484 injuries, 96 were fatal. The sample, consisting of cases that occurred between 2012 and 2013, consisted of 2,113 male and 371 female subjects; the overall average age was 33.2 years. The authors found that most of the injuries occurred in agricultural, food and tobacco industrial, chemical, metal, electronic and non-electrical work environments. With regard to the use of safety equipment this aspect was not specified; the toxicological analyses were only focused on cases of gas poisoning (22).

The retrospective study conducted by Nazanin et al. focuses on a 10 year period of occupational accidents, with a total of 207,604 cases, of which 1,079 were fatal (0.52%), with a predominance of the male gender and a prevalence of fatalities in agriculture and industry, within the work environment, compared to accidents outside it. The authors also noted that in 26.7% of the cases the workers were not wearing personal safety equipment (23).

Pouradeli et al., in a cross-selective study, analysed a case history of 2,228 accidents, of which 263 were fatal. 2,181 subjects were male, 47 female, with an overall average age of 34.5 years. The toxicological analyses conducted revealed that poisoning by unspecified toxic substances occurred in six cases (24).

#### 3.2.3 Group 3—average HDI (India)

##### 3.2.3.1 India

As far as South-East Asia is concerned, the authors selected the study conducted by Chittaranjan et al. in India, reporting a singular category of work-related injuries, occurring due to electrocution, taking into account that most studies already mention chemical poisonings and that the actual number of injuries due to mechanical trauma is to be considered underestimated. The study cited analysed 428 cases of which 90 were work-related (21.03%) and in which 376 were men, 52 women, with an average age of 19.8 years. In no case was the use of PPE investigated (25).

#### 3.2.4 Group 4—low ISU (Tanzania)

##### 3.2.4.1 Tanzania

The retrospective study conducted by Shewijo et al. found that, in a sample of 4,578 cases of occupational accidents, 460 of which were fatal, the main cause was polytrauma occurring in the context of public and private industrial environments, where there was no information on the correct use of safety equipment and where the workers were predominantly men (3703). In their study, of interest for preventive purposes, the work shift was also taken into account. Most of the adverse events occurred in the manufacturing sector, followed by the agricultural and forestry sectors, and the construction sector without, however, going into the application of PPE (26).

#### 3.2.5 Personal protective equipment legislation

As mentioned above, for each group selected on the basis of HDI, the use of personal protective equipment for occupational safety was investigated. The sources analysed by the authors were the National Institute for Occupational Safety and Health (NIOSH), the International Labour Organisation (ILO), the Occupational Safety and Health Administration (OSHA) and the local government websites of the countries involved in the study.

**Figure 6:**
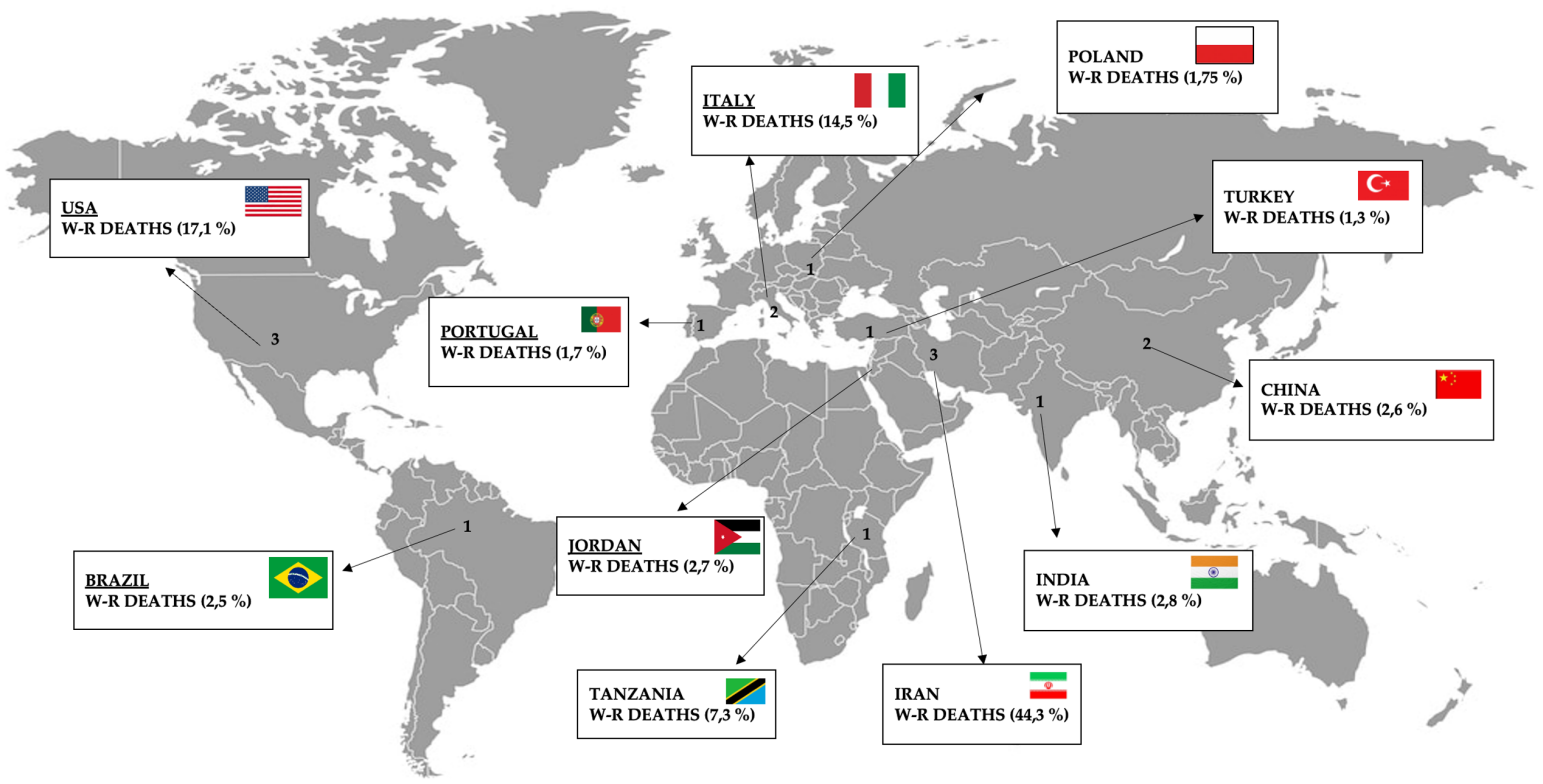
Representative figure illustrating the percentage of work-related (W-R) deaths according to the articles included in this review.

As enacted in Article 16—Convention on Safety and Health at Work, enacted in Geneva in 1981 (27, 28), employers have duties regarding the provision and use of personal protective equipment (PPE) at work. PPE is equipment that will protect the user from the risk of accidents or adverse health effects. It can include items such as safety helmets, gloves, eye protection, high visibility clothing, safety footwear, safety harnesses and respiratory protective equipment (RPE). By 2023, the convention had been ratified by 78 states, 17 of which had also ratified the 2002 Additional Protocol. Analysing the current member states, with reference to the studies selected in the study, it can be seen that of the Groups selected on the basis of the HDI, in Italy the principles of the 1981 Convention and subsequent acts issued by the ILO are reflected in the Safety Consolidation Act (D.L. 81/08), in the United States it has been implemented since 2002 (i.e., after the corrigendum drafted with NIOSH), in Portugal it has been active since 1985, in Turkey since 2005, in Poland it is not active. Among the Group 2 countries, China implemented the Convention in 2007, Brazil in 1992, in Jordan it is not active, in Iran since 1995. In India, a country with a medium HDI (Group 3), the Occupational Safety and Health Convention has not yet been implemented and this may account for the large number of accidents observed; in Tanzania (Group 4—low HDI), it was found that OSHA has concluded agreements on the correct use of PPE with each of the local ministries of the countries selected in the study, including Tanzania, a country classified as low HDI (29) (Figure 6).

## 4 Discussion

Accidents at work are an inherent problem in the performance of work itself. Over the years, attempts have been made to deal with them by investing, in terms of research into the evolution of work activities and any new emerging risks, in the design and application of new individual safety devices, together with increasingly specific prevention measures for each work activity (2).

These events can be related to ergonomic factors as well as to incorrect behaviour and unintentional errors.

Ergonomic factors play a significant role in occupational safety, arising mainly from the interaction between workers and various elements of the work system. Common problems include adopting incorrect postures, performing repetitive movements without ergonomic considerations, lifting and handling heavy loads without mechanical assistance, and working in environments unsuitable for optimal performance due to factors such as poor hygiene, uneven flooring, inadequate lighting, and obsolete or poorly maintained machinery (30).

When discussing behaviours or errors that can result in injuries, it is helpful to reference Rasmussen’s classification of errors. This classification identifies various types of errors, including lapses which result from practical oversights, lapsus which stem from habit or established memory, rule-based errors caused by misapplication of rules despite correct situational identification, and knowledge-based errors arising from incomplete or incorrect understanding of the situation (4).

The review work carried out by the authors analysed cases of fatal and non-fatal occupational injuries from the demographic aspect; in this regard, the selected studies were stratified according to the group to which each country belongs in terms of the Human Development Index, an indicator of macroeconomic development that takes into account life expectancy, level of education and income. The division of countries according to the Human Development Index (HDI) was chosen because it allows for an understanding of any disparities between countries, and thus guide the development of policies aimed at improving working conditions and employment security based on the specific needs of each country group. In addition, HDI can be used as an indicator to assess the impact of policies and interventions on employment security in different categories of countries. This makes it possible to monitor progress in reducing occupational accidents and improving working conditions over time.

The review work made it possible to confirm, from a comparative statistical point of view, the previous studies carried out on individual countries, which, in fact, form the basis for continued study and growth based on the reactive analyses conducted.

From a bibliographical point of view, the screening phase carried out to select the studies, depending on the inclusion criteria chosen, made it possible to find that, beyond what was then analysed, there are innumerable causes of accidents that also lead to actual occupational diseases, especially cardiovascular and pulmonary, especially in jobs that are common to domestic life, such as cooking and maintenance work, which, from an occupational point of view, are subject to health surveillance (3).

Once the selection was made, the analysis focused on assessing the number of injuries, particularly fatal ones, the geographical areas and the ways in which they occurred, and whether the use of personal safety equipment was investigated. Indeed, PPE is one of the most important preventive elements in safety, and reconstructing the dynamics of injuries, assessing whether or not they were used, is important both to evaluate their effectiveness and to recommend their use (31).

The gender and age distribution of the subjects is heterogeneous, although not all studies have investigated these aspects in depth.

With regard to the sectors mainly involved in occupational accidents, it has been found that industries are still the main accident location; this could be linked to both environmental and individual factors.

It should be noted that industries are often classified into broad sectoral groups, such as manufacturing, construction, healthcare, agriculture and services, and others. Each of these sectors is recognised for its unique contribution to the economy and society.

Each production area or subsector may expose workers to specific risks. For example, the production of chemicals requires protection against hazardous substances, while construction workers must be protected against falls and flying objects.

As industries adopt new technologies, the nature of the work and associated risks evolve, leading to new requirements for PPE as well. For example, the advent of nanotechnology in manufacturing has introduced nanomaterials, which require specialised respiratory protection.

Demographically, the data in the scientific literature are in line with the authors’ findings in the systematic review procedure, noting that, in percentage terms, Iran is the country with the highest work-related fatality rate (44.3%), followed by the United States (17.1%), Italy (14.57%), Tanzania (7.27%) and, with percentages of less than 3%, the other countries examined. These data show a correlation with the industrial and macroeconomic development of the demographic areas of which they are representative, with a lower incidence of injuries in richer countries (high, medium-high HDI), where there is also more investment in safety. The figure for Tanzania (which falls in the group of low HDI countries) could be confusing as a result of underreporting of work-related injuries, and the reduced development in terms of the legality of work, which is often carried out without actual contracts (26).

In line with what has been observed, what is lacking in the study of most of the reviews carried out on occupational injury cases is an in-depth assessment of the medical and medical history of the subjects as well as compliance with the regulations concerning safety in the workplace or the consumption of alcohol and drugs - to be understood as acute consumption during, or in the immediate vicinity of, working hours. More specifically, with regard to the type of sector most involved in fatal and non-fatal occupational accidents, it was observed that in low-income countries (India) or developed countries with significant agricultural areas (China), most accidents are attributable to the performance of agricultural activity, both in terms of mechanical trauma and poisoning, mainly through the use of pesticides such as organophosphates and carbamates, substances with muscarinic, nicotinic and neurotoxic action. The joint observation of a lack of analysis of the actual use of personal safety equipment supports the hypothesis that in post-event investigations, this is a data point to be investigated for preventive purposes.

In terms of health surveillance and the use of individual safety equipment, in fact, in only two out of 16 studies was attention paid to the correct use of PPE, a discouraging fact, bearing in mind that the sample, in terms of causes and methods of occurrence of accidents, was fairly homogeneous and that the legislation in force in the individual countries provides for the application of similar measures country by country, with specific coding nomenclature (for example, in India the IS (Indian Standard) Codes for PPEs and Safety Equipment is in use, which include, as an example IS code of Safety Belt and Full body Harness—IS 3521: 1999. IS Code for Industrial safety helmet—IS 2925: 1984).

Certainly the spread of occupational safety regulations, greater industrialisation and mechanisation, including in agriculture, are elements that have contributed to reducing the number of fatalities but not disabling accidents (32). In fact, the industrial and agricultural sectors still account for the highest number of fatalities per year. These data correspond to those discussed in this systematic review and underline that work-related injuries and deaths are still a major global public health problem.

The evidence above demonstrates that there is no globally uniform procollo for verifying the use of Personal Protective Equipment (PPE), which is crucial to ensuring consistent standards of workplace safety worldwide. Such a protocol would establish clear and uniform procedures for the proper use of PPE, thereby reducing the risk of accidents due to misapplication or inappropriate use of PPE. In addition, uniform regulation would increase awareness among workers and employers of the vital importance of PPE for personal protection. Such awareness could, in turn, help reduce exposure to hazardous substances, prevent occupational injuries and illnesses, and improve the overall health and safety of workers worldwide. A uniform global protocol would also simplify the regulatory compliance process for companies operating on an international scale, facilitating compliance with legal requirements for PPE use in different national contexts. In addition, promoting the exchange of knowledge and best practices across countries and sectors would be an additional benefit of adopting a uniform protocol. This would encourage the continuous improvement of policies and procedures related to the use of PPE, ensuring that they are always at the forefront of worker safety and protection.

Finally, an important aspect is the inequality between countries in terms of prevention and treatment systems; this aspect has been addressed by WHO, as in terms of outcomes it is important to take into account not only the potential fatality of injuries, but also the disabilities that can result from poor care. The latter, in particular, particularly affect overall global health costs, with estimates of figures exceeding development spending (33).

A recent study analysed precisely the economics of noncommunicable diseases (including those caused by trauma) in developing countries, confirming existing gaps in terms of investment in prevention (34).

## 5 Conclusion

The correct use of appropriate PPE is vital for worker safety and can be a determining factor between accidents and safety. Indeed, several studies had indicated a significant association between the lack of PPE use and occupational accidents (32, 35, 36).

The low level of awareness of their use, inadequate use or not using them at all, are elements that have, over time, significantly contributed to increasing the risk of work-related injuries. Studies also reveal that many workers, although they use PPE, remove it during their work activity, arbitrarily and with little knowledge of the potential consequences related to the ‘violent cause’ element that characterises workplace accidents.

The selected studies offer a snapshot of the severity of the problem, of the lack of investigation into the use of PPE; however, in the studies reviewed by the authors, the focus was on fatal injuries; to this must be added the high number of disabling injuries, which also represent a high impact on healthcare costs.

Significant differences between the types of accidents in low-income and Western countries confirmed the data from previous literature. In rural areas and Asian countries, most work-related deaths occur mainly due to problems with toxic substances, to a lesser extent with machinery and electrical equipment and, where reported, due to a lack of safety devices. In Western countries, as expected, industrial machinery and construction play a major role.

In conclusion, the bibliographical screening carried out during the selection of the articles to be included in the study showed that information relating to socio-demographic, anamnestic and psychopathological factors was often lacking; furthermore, taking into account the inclusion and exclusion criteria, not all geographical areas were investigated, making the results not fully representative of the epidemiological scenario. The articles included in the study were deficient in the in-depth study of the use of individual safety devices, also in relation to the specific task, not providing fundamental information in terms of their correct and constant use, and their effectiveness.

It is to be hoped that future research will pay greater attention both to the correlation between the socio-demographic and anamnestic characteristics of workers, and to the careful verification of the correct and constant use of personal protective equipment, a pivotal element in better highlighting their effectiveness in relation to risk factors, favouring the implementation of specific prevention programmes.

All this would place greater emphasis on appropriate information and training campaigns to counter the phenomenon of occupational accidents, which is attributable, at least in part, to the lack of awareness among workers.

### 5.1 Strengths and limitations of the study

#### 5.1.1 Strengths

Comprehensive geographical and sectoral analysis: The study provides a broad analysis of occupational accidents in different countries, focusing on different sectors. This approach allows a broad understanding of occupational accidents in various contexts.

Systematic review procedure: The authors followed a systematic review procedure to collect and analyse the data, which is in line with rigorous research standards and contributes to the reliability of the results.

Demographic analysis: The study includes a demographic analysis of those involved in occupational accidents, offering insights into age and gender distribution that can inform targeted prevention strategies.

#### 5.1.2 Limitations

Heterogeneous gender and age distribution: Although the study addresses the distribution by gender and age, the lack of depth in this analysis may mean that important nuances regarding how different demographic groups experience work-related injuries are lost.

Lack of in-depth assessment: The absence of a detailed assessment of medical and medical history, compliance with safety regulations and alcohol and drug use limits the study’s ability to provide a comprehensive understanding of the factors contributing to work-related injuries.

Absence of a standardised protocol for analysing accidents: The study highlights the absence of a standardised protocol for analysing accidents at work, which could improve both preventive measures and responses to accidents.

The strengths and limitations suggest that while the study offers valuable insights into workplace injuries and the role of PPE, there are opportunities for future research to address its limitations. Future studies could benefit from incorporating a broader analysis of safety measures, performing more detailed demographic analyses, and developing standardised protocols for analysing injuries.

## Data availability statement

The original contributions presented in the study are included in the article/supplementary material, further inquiries can be directed to the corresponding author.

## Author contributions

GM: Writing – original draft, Writing – review & editing. SF: Writing – review & editing. FP: Writing – original draft. AA: Writing – review & editing. EC: Writing – original draft, Writing – review & editing. SM: Writing – review & editing, Methodology, Supervision, Visualization. AC: Writing – review & editing, Methodology, Conceptualization, Visualization.
